# Autistic Adult Online Interoception Feasibility Pilot Program

**DOI:** 10.1155/oti/4437761

**Published:** 2026-06-18

**Authors:** Sophia Graziosi, Emily Oliver, Victoria Arline, Kelly Mahler, Karen Ratcliff, Loree Pryor, Claudia Hilton

**Affiliations:** ^1^ Occupational, Therapy Department, Children′s Therapy Center, Inc., Texas City, Texas, USA; ^2^ Occupational Therapy Department, Playworks Pediatric Therapy Services, Dallas, Texas, USA; ^3^ Occupational Therapy Department, The University of Texas Medical Branch at Galveston, Galveston, Texas, USA, utmb.edu; ^4^ Occupational Therapy Department, Elizabethtown College, Elizabethtown, Pennsylvania, USA; ^5^ Occupational Therapy Department, Brazosport Independent School District, Brazosport, Texas, USA; ^6^ Community Engagement and Education Department, The University of Texas Medical Branch at Galveston, Galveston, Texas, USA, utmb.edu; ^7^ Occupational Therapy Department, School of Health Professions University Blvd., The University of Texas Medical Branch at Galveston, Galveston, Texas, USA, utmb.edu

**Keywords:** alexithymia, anxiety, interoception, quality of life

## Abstract

Interoception differences are known to co‐occur with autism and result in difficulty recognizing and addressing internal signals, which can produce barriers to daily living and quality of life (QoL). Limited supportive services have addressed these needs in autistic adults, and occupational therapists are well prepared to provide interventions to address these challenges. The objectives of the study were to: (1) examine feasibility of an 8‐week online interoception program for autistic adults and (2) examine quantitative changes after participation in the program. The program was adapted from the *Interoception Curriculum* for 10 autistic adults. Outcome measures included standardized questionnaires for interoception, anxiety, alexithymia, QoL, and self‐designed questionnaires for program evaluations. Researchers were supported throughout the process by a paid autistic occupational therapist to guide development of the research question, methodology, and adaptation of support materials to incorporate lived experience and community engagement. Preintervention and postintervention scores were compared for interoceptive awareness, alexithymia, anxiety, and QoL, which showed insignificant improvements in all areas with a range of small to large effect sizes over the 8 weeks. Participants reported positive feelings about the program and would recommend it to others. Results support the feasibility of an online interoception program for autistic adults.

**Plain Language Summary**

Autistic people sometimes have difficulty understanding some of their own feelings such as thirst or pain. These feelings are part of a category of sensations called interoception. These difficulties can get in the way of people being able to do regular daily activities and to enjoy life. Very few programs are available to help with these needs in autistic adults. The 8‐week program for this study was carried out online. Ten autistic adults between 25 and 48 years old participated. Scores for interoception and other related areas improved. Participants reported positive experiences and would recommend the program to others. Improved scores in this small study tell us that using this program could help autistic adults with this type of difficulty. Future larger studies are needed to examine the effectiveness of this type of program.

## 1. Autistic Adult Online Interoception Feasibility Pilot Program

Our internal body signals underpin our physiological and mental states, which function as cues to guide our behavior [1]. Sensing and interpreting these changes are linked to our ability to regulate and engage with the world and are essential for survival [1]. We can access this important information through interoception [2, 3]. Interoception allows for processing and interpreting internal, physiological feedback from the body to guide motivational and behavioral responses such as eating, hydration, pain management, elimination, and stress management [4, 5]. Interoception includes sensing, interpreting, and integrating signals originating from within the body across both conscious and unconscious levels of processing and includes multiple areas of the brain in the neural network [3, 6]. A recently expanded conceptualization presents interoception as a complex multidimensional system, with bidirectional features between the brain and the body [7]. This interplay is necessary to maintain homeostasis and to respond adaptively to the changes in a person′s internal and external environments. Interoception differences experienced by adolescents and adults may reduce understanding of internal bodily sensations such as thirst, pain, anxiety, fatigue, or illness [8]. Individuals with interoception differences can experience difficulty completing daily occupations including self‐care and management of health and emotional awareness and regulation [7]. Findings from several studies suggest that interoception ultimately affects emotional awareness and emotional regulation in children [9] and adults [10, 11]. All of these are important for identifying occupational therapy strategies to increase occupational performance, participation, and engagement [12].

Interoception differences are related to several other human traits as described below and interoception may be a possible occupational therapy intervention focus when addressing other concerns. Relationships between sensory processing and interoception have been found in several studies. Grist et al. [13] found significant positive predictive relationships between sensory processing and interoceptive awareness variables in children. Sensory processing differences were correlated with interoception differences in an adult study [14], and anxiety, alexithymia, and quality of life (QoL) were shown to be related to interoception in several other adult studies [15, 16]. Understanding relationships between these factors is important for increasing overall knowledge of interoception and understanding how interoception affects autistic adults. This understanding may be a vital step toward improving the overall well‐being and occupational engagement of autistic adults.

Autistic individuals often experience interoception differences compared with neurotypical child and adult peers [17]. Autistic adults may exhibit both hypoawareness and/or hyperawareness of interoceptive stimuli [18, 19]. Hypoawareness is described as decreased or muted awareness of interoceptive sensations, such as hunger, thirst, pain, illness, or other internal signals, and hyperawareness is described as the opposite, or hypochondria, where individuals have an intense awareness of internal body signals [20]. These differences in awareness, both more and less, can increase the risk of disordered eating patterns, ineffective self‐care practices, and participation and can also minimize seeking timely help from healthcare professionals [21]. For example, reduced sensations of internal pain from a digestive problem may result in delayed attention by medical professionals when problems exist, whereas heightened feelings of muscular pain during movement may limit the person′s interest in participating in sports or other moving activities. In addition, some autistic adults may over‐hydrate because of reduced awareness of sensations indicating that thirst has been satisfied, which can increase the incidence of seizures, a common symptom experienced by autistic adults [22, 23]. The degree of interoceptive differences varies among autistic adults and the exact presentation, the pattern of these differences, and differences between children and adults, are unclear [20, 24]. Differing findings seem to be related to whether interoceptive accuracy, interoceptive sensibility, or interoceptive awareness are examined [25]. Interoceptive accuracy examines performance on objective tests, such as heart rate accuracy, whereas interoceptive sensibility examines self‐reported beliefs concerning one′s own interoception. Interoceptive awareness examines the amount of agreement between accuracy of performance and self‐assessed performance. In a systematic review and meta‐analysis, heart rate interoceptive accuracy was not found to be significantly different for autistic adults compared with neurotypical adults in all five studies reviewed [26]. Whether differences are present in autism for other interoceptive dimensions across age groups was unclear.

Interoception differences are prominently featured in diagnostic classifications of anxiety disorders across the lifespan [27, 28]. Though evidence is mixed, anxiety is often considered to be the prototypical interoceptive disorder and an important occupational therapy intervention target for clients with anxiety issues [29, 30]. Approximately 53% of autistic adults meet the criteria for anxiety disorder, contrasted with 10%–15% of nonautistic adults [31]. Symptoms include excessive feelings of worry, fear or unease that are difficult to regulate and interfere with daily functioning in children and adults [32]. Anxiety results from having difficulty regulating the feelings of worry, fear, and unease and may suggest a need for emotional regulation support. Co‐occurring anxiety in a person with autism can amplify autistic differences such as reduced social skills, resistance to change, and increased repetitive behaviors. Persons with anxiety disorders often report heightened interoceptive sensations. Despite an increased prevalence of anxiety among autistic adults, mental health is not always thoroughly addressed, nor are there many evidence‐based interventions adapted to the emotional regulation needs of autistic adults [33]. The emotion dysregulation model is based on evidence suggesting that adults with anxiety disorders experience differences in emotion regulation. Generalized anxiety disorder (GAD) is marked by experiencing emotions quickly, with high intensity, and the emotional reactivity in GAD makes emotions difficult to regulate for people of all ages [34]. Difficulties experienced from anxiety can be further complicated by interoception differences in autistic individuals.

Alexithymia is another condition related to interoception. It is defined as difficulty identifying and describing how one feels and its relation to internal body sensations [35] and often co‐occurs with the diagnosis of autism [5, 16]. Differences in interoception have been proposed as the core reason for alexithymia [36]. When an individual is not aware of bodily signals or misinterprets internal bodily signals, they miss reliable information needed to inform emotional judgment [33]. Several researchers postulated that emotional processing differences in autistic adults may partially be due to interoception differences [1, 37]. Support for this was added by another study, finding that visceral perception played a role in the experience of the intensity of emotion [38]. Although the link between interoception and alexithymia is generally supported across studies, more research examining causality is needed [20, 24]. Jakobsen and Rigby [39] found evidence suggesting that alexithymia is associated with differences in both bottom–up and top–down sensory processes that affect the processing and regulation of emotions in adults, important relationships when choosing sensory processing intervention options for occupational therapy. Their findings also suggested that individuals with different alexithymia subtypes may differ in other areas, including early life experiences, mental health outcomes, and susceptibility to various personality disorders.

Alexithymia has been reported in half of autistic adults, which is five times that of the general population [40] and was commonly but not universally seen in autism in a meta‐analysis of 15 studies of autistic children and adults [41]. This supports a growing body of evidence that co‐occurring autism and alexithymia represent a specific subgroup in the autistic population who may have specific clinical needs. Emerging evidence indicates that alexithymia is both a cause and consequence of autistic behaviors [37]. Several studies have examined the relationship between alexithymia and other common concerns experienced by autistic adults. Hassen et al. [42] found alexithymia to be correlated with emotional regulation, and Morie et al. [43] found alexithymia and emotional regulation to be mediators between autistic features and depression and anxiety. Difficulties in identifying feelings, a specific alexithymia factor examined in this study, were the strongest predictor for depressive symptomatology in autism in one adult study, outweighing autism traits and other alexithymia factors [44]. In another study, higher alexithymia traits predicted lower levels of social competence when comparing autistic and nonautistic children [45]. Findings from studies examining these relationships attest to the importance of expanding this knowledge so that effective occupational therapy interventions can be developed.

QoL may also be related to interoception. It is defined as a person′s perception of their position in life and general well‐being, including emotional, social, physical, and environmental aspects of their lives (WHO 1998). Dunn et al. [46] stated that interoception contributes to an overall sense of wellbeing. Autistic people have been reported to have a much lower QoL compared with people without autism across the lifespan [47]. When separating by age, behavior problems, and leisure activities were associated with the QoL for the majority of autistic adults [48]. Examining specific aspects of QoL, Lin and Huang [15] found that autistic adults demonstrated reduced scores compared with norms in all four QoL domains: physical health, psychological health, social relationships, and the environment, with significantly lower scores in psychological health and social relationships. In a later study, Oakley et al. [49] found that although autistic adults generally scored lower than comparison individuals, 36%–71% of autistic individuals did not report a poor QoL across the QoL domains when subjective measures were used. Notably, both studies [15, 49] concluded that low QoL was associated with social communication and mental health challenges such as depressive and anxiety symptoms, both of which have been linked to interoception and alexithymia.

As autistic individuals enter adulthood, a dearth of appropriate supportive services exists to meaningfully address their changing needs [50]. Despite many autistic adults reporting that interoception differences are impacting many areas of life including emotional awareness, emotional regulation, and QoL, minimal research has been published on interoception programs for autistic adults [4, 51]. Use of the *Interoceptive Curriculum* has been studied in pediatric populations [9, 52] but not in an adult population. Aims of this study were to (1) examine the feasibility of an 8‐week online interoception program for autistic adults and (2) examine quantitative changes after participation in the program. The intent was to examine whether it is feasible to conduct an online program to address interoception in autistic adults while determining the appropriateness of the evaluation tools used and testing the intervention on a small number of participants before conducting a larger study using the intervention [53]. Both qualitative and quantitative data were used to triangulate the findings and substantiate the value of the outcomes. It was hypothesized that following participation in the study, participants would report decreased anxiety and alexithymia, and increased interoception and QoL.

## 2. Methods

### 2.1. Intervention

This prepost pilot study measured the feasibility and changes observed after an 8‐week interoception program on interoception, anxiety, alexithymia, and QoL in autistic adults. It was approved by the Institutional Review Board at the University of Texas Medical Branch (#23‐0019). STROBE guidelines were used to guide the study [54]. This study received no funding to support its completion.

### 2.2. Participatory Methods

To increase the meaningfulness of the project, researchers were supported throughout the research process by an occupational therapist with a professional autism diagnosis who was financially compensated to guide development of the research question, methodology, and adaptation of support materials. Each step of the process was vetted through this advisor before the final versions of the conceptual development, recruitment, outcome measures, and intervention were finalized.

### 2.3. Recruitment Strategy

Study participants were recruited through word‐of‐mouth, email, and social media platforms such as Facebook and Instagram. Recruitment occurred over a 2‐week time period during which potential participants were asked to complete an online interest survey. Potential participants were also able to directly contact researchers via email for more information. International participants received information about the program through the same social media posts as US residents and contacted researchers via email if they were interested in participating. Verbal consent for participation in the program was given during initial Zoom meetings with individual participants, and all gave full consent. After providing consent, each participant was sent program materials via email, including information on planned content, a schedule with session times, workbook materials, and assessments. Participants were not paid or otherwise compensated for their participation in the study.

### 2.4. Inclusion and Exclusion Criteria

Eligible study participants were 25 years of age or older, self‐reported as autistic, were able to read and sign their own consent form and had access to reliable internet and the Zoom application to be able to participate in the online sessions. To reduce subject burden, the participants′ ability to read and sign consent forms was used to ascertain level of functioning in lieu of adding an additional functional test. Age criterion was set to target participants who would be old enough to participate in the workforce and beyond the age of neuroplastic pruning that occurs in young adulthood [55]. Exclusion criteria consisted of individuals below the age of 25 years and those who did not have an autism diagnosis. Non‐English speakers were not included because the researchers were only English speaking and there was no budget available for hiring an interpreter.

### 2.5. Procedures

#### 2.5.1. Overview

Participants attended an 8‐week program consisting of weekly 60‐min online group sessions, and homework practice activities via Zoom. Optional individual coaching sessions no longer than 60 min via Zoom or email were also provided in the format preferred by the participant. Prior to the first session, participants were sent a workbook to use throughout the program. This workbook contained the purpose and messages of the program as well as visual guides for in‐session and homework activities. All activities included in the workbook were from *The Interoception Curriculum* [56] with some adaptations or additions based on suitability for the online program format and for adult participants, which will be described below. See Appendix 1 for examples from workbook. Other interoception programs have been developed for children, but the most comprehensive found for adults is the *Interoception Curriculum,* which has a range of useful supplemental materials that have shown promising effects in a pediatric population [9, 52, 57] and can be adapted for adults.

The *Interoception Curriculum* provides a step‐by‐step framework to guide discovery about individualized body‐emotion‐action connections [56]. It uses strategies called *Interoceptive Awareness Builders* (IABs), which are forms of adapted body mindfulness, or the purposeful nonjudgmental focus on how the body feels in the present moment. The use of body mindfulness is well supported for enhancing interoception in certain adult populations [58–61] but has not been explored with autistic adult participants. Quadt et al. [16] used interoceptive training to reduce anxiety in autistic adults, specifically heartbeat detection, a component of interoceptive accuracy.

Two main IABs were used throughout the current program: *descriptor menus* and *focus area experiments* [56]. These *IABs* were incorporated throughout the online sessions and were also provided as structured homework activities so that the participants had extensive opportunities to practice noticing and describing body sensations across a variety of contexts. The *descriptor menu* is a visual menu of words that could be used to describe the way the body part felt. The *focus area experiments* are aimed at evoking sensations comfortably and playfully within the body part of focus to provide practice noticing and describing how specific body parts felt. Providing robust, explicit practice opportunities is based on theoretical work from Myles et al. [62] and Ericsson [63]. Rather than focus on “fixing negative feelings,” the IABs seek to nonjudgmentally explore a variety of bodily reactions and find experiments or actions that produce comfortable body signals, similar to the work by Seligman [64] that focuses on generating more positive emotions. For this online program, the *Interoception Curriculum* was consolidated into an 8‐week format with two to three body parts covered per session. The original curriculum focused on one body part, each session with longer focus provided if clients required more practice.

#### 2.5.2. Weekly Online Group Sessions

Each online group session was led by two to three doctoral occupational therapy students with oversight by two faculty advisors. To provide a predictable learning environment appropriate for the online group of adults, researchers created and presented weekly PowerPoint slides containing a session schedule and visual guides to activities used during the session. See Appendix 2 for example of PowerPoint slides. Each weekly group session followed the same format and focused on noticing and describing sensations in one or two specific body parts (e.g., hands, feet, skin, mouth, eyes, heart, and brain). To start each session, participants and researchers worked together to create a *descriptor menu* followed by *focus area experiments* [56]. At the conclusion to each session, the homework assignments for the upcoming week were briefly reviewed.

Throughout the sessions, the approach was to invite participation and use interactive, hands‐on learning to foster discussion between researchers and participants. Participants and researchers interacted via a mix of speaking into the microphone and/or using the chat feature on Zoom. Emphasis throughout the sessions was on curiosity and helping each person discover their unique internal experience (with repeated emphasis on inner diversity and the fact that there were no wrong interoception answers). All responses were validated and honored as correct by the facilitators.

#### 2.5.3. Homework Activities

Further adaptations for the online and adult participants included daily homework activities that were structured in the workbook and reviewed at the end of each session. These activities utilized naturally occurring *focus area experiments* by inviting participants to select a daily activity that might evoke interoceptive sensations, such as watching television or putting on their shoes, and notice how a specific body part felt during that activity (e.g., hands or stomach). See Appendix 3 for examples of experiments. Participants were asked to record their observations in a designated spot in the workbook. Additionally, a second homework activity included ideas for more *focus area experiments* for each body part covered during the weekly online session. For this, the participants were invited to record their observations in the workbook. To support the homework process, researchers emailed each participant the *descriptor menus* generated during the online sessions to provide language support during the *focus area experiments* if needed. Participants were also asked to list feel‐good activities learned from each session that they could use later to improve their positive sensory experiences.

### 2.6. Optional Coaching Sessions

Another adaptation of this program to support connectedness between researchers and participants was to add optional coaching sessions. Each week, optional individual coaching sessions were offered to participants to help with the homework activities and/or offer any other interoception‐based support. Participants could choose a coaching session on Zoom or conversing over email. Coaching sessions were participant‐driven and focused on reviewing the weekly session topic, clarifying unclear information, and completing *Focus Area Experiments* together. Coaching has successfully been used with autistic adults. Increases in academic performance, decreased rates of expulsion, and high levels of satisfaction resulted from coaching used with autistic college students [65]. Additionally, virtual coaching was found to be an important complement to standard treatment for adolescents and young adults with attention deficit and autism spectrum disorders [66, 67].

### 2.7. Evaluation Methods

#### 2.7.1. Feasibility

Questions addressing feasibility included whether this type of program would be acceptable to adults, whether the online format would be acceptable to participants, whether they could gain knowledge from this format, whether they would sustain attendance for the 8 weeks of the program, and whether they would find the coaching sessions valuable for their learning. Criteria for feasibility thresholds were set at being able to recruit at least 10 participants, being able to retain at least 80% of participants for the duration of the program, quantitative outcomes showing some degree of improvement from baseline to end of program, and 80% of participants stating that they would recommend the program to other autistic adults [68].

An attempt was made to utilize assessments with limited questions to reduce the burden on participants. Quantitative data were obtained during Weeks 1 and 8 of the program from the Interoception Sensory Questionnaire (ISQ‐8), eight‐item general alexithymia factor score (GAFS‐8), general anxiety disorder (GAD‐7), and World Health Organization Quality of Life—BREF (WHOQoL—BREF) with the autism QoL module. Prior to starting and after completing the program, participants were emailed a link to a Google survey containing the ISQ‐8, GAFS‐8, GAD‐7, and WHOQoL—BREF.

A 20‐question program evaluation in the form of a Google survey was requested from participants at the midterm (Week 4) and end of the program (Week 8). Questions consisted of Likert scale and open‐ended questions, which were written by researchers to gain participants′ perceptions of the structure of group and coaching sessions, their perceived improvement, and instructor appraisal. The questions were reviewed and approved by the autistic occupational therapist advisor. Examples included “I will use some of the strategies I learned in this program,” “The materials used in the weekly sessions were useful for improving my understanding of interoception,” “What was the most helpful part of this program?” and “What changes would you recommend?” See Appendix 4 for the list of questions. After the Week 4 program evaluation, researchers implemented recommended adjustments that were feasible for the rest of the program.

#### 2.7.2. ISQ‐8 (Interoception)

The ISQ‐8 is an eight‐question, self‐report assessment that was modified by Suzman et al. [69] from the 20‐item ISQ by Fiene, Ireland, and Brownlow [70]. Answers on the ISQ‐8 are scaled from “not true at all of me” (score = 1) to “very true for me” (score = 5). Results on the ISQ‐8 are based on *T*‐scores and *Z*‐scores ([70]; *m* = 50, sd = 10). Higher *T*‐scores indicate decreased interoceptive awareness. The ISQ‐8 has high score reliability (alpha = 0.90), is valid for both autistic adults and adolescents, has a unidimensional structure, and has excellent model‐data fit [71].

*GAD-7* (anxiety). The GAD‐7 is a 7‐question screening assessment that quantifies the severity of anxiety symptoms experienced by an individual over the past 2 weeks [72]. The response options for each question are not at all (0), several days [8], more than half the days [73], and nearly every day (3). Total scores are categorized into minimal anxiety (0–4), mild anxiety [36, 40, 44, 50, 74], moderate anxiety [1, 29, 31, 48, 51], and severe anxiety (15–21; [75]). The GAD‐7 has good reliability (alpha = 0.82–0.91), internal consistency, convergent validity, and discriminant validity among anxious and more general psychiatric populations [72].

#### 2.7.3. GAFS‐8 (Alexithymia)

The GAFS‐8 is an eight‐item self‐report assessment used to determine an individual′s level of alexithymia and was adapted by Williams and Gotham [76] from the 20‐item Toronto Alexithymia Scale [77]. Answer choices range from strongly disagree (score = 1) to strongly agree (score = 5). *T*‐scores are categorized as: low alexithymia (< 40), average alexithymia [6, 14, 15, 19, 24–26, 28, 30, 34, 37, 41, 43, 49, 52, 56, 57, 61, 62, 72, 78], high alexithymia (> 60), and very high alexithymia (> 70). Previous research has shown high score reliability (alpha = 0.7; [76]) and strong nomological validity. Questions are written at a reading level appropriate for younger individuals, people with less education, or less cognitively able respondents.

#### 2.7.4. WHOQoL‐BREF (QoL)

The World Health Organization Quality of Life Measure (WHOQoL‐BREF) was used to measure participant QoL for this study. The WHOQoL‐BREF is a 26‐item self‐report questionnaire that assesses the QoL through four domains: physical, psychological, social, and environmental [79]. It uses a 5‐point Likert scale to obtain an overall QoL score with scores ranging from 0 to 100, with higher scores indicating a higher subjective QoL. The WHOQoL‐BREF has good reliability (alpha = 0.71–0.82 for autistic adults; [80]) for the total score and structural validity, as well as criterion, convergent, divergent, and discriminant validity in previous studies assessing QoL among autistic and nonautistic individuals.

### 2.8. Data Analysis

Trustworthiness. Steps to support trustworthiness when obtaining program evaluation qualitative data included efforts to support credibility, dependability, confirmability and transferability [81]. Credibility was supported through the prolonged engagement over the 8‐week program with participants and the continued opportunity for participant feedback during the sessions and the in‐depth accounts of the participants′ experiences. Reflexivity was encouraged among the researchers at weekly debriefing meetings following each online session, discussing personal assumptions, biases and their roles in the research process. Member checking with the participants was attempted during the educational and coaching sessions, encouraging sharing of their personal experiences with the interoceptive activities and with situations experienced outside of the sessions. Dependability was supported through the mentoring by faculty members and the autistic consultant and the careful development of the weekly program sessions using materials from *The Interoception Curriculum*. Confirmability was supported by the debriefing among the study participants after each interoception activity, during the coaching sessions, and by the debriefing after each session among the researchers. Transferability was supported through the development of a workbook for participants, which was developed based on *The Interoception Curriculum* and included the weekly presentations and homework assignments. Thick and detailed descriptions of participant experiences were encouraged during each of the online and coaching sessions.

Program evaluation responses were reviewed and suggested changes were implemented when possible. The ISQ‐8, GAD‐7, GAFS‐8, and WHOQoL‐BREF were scored using the online scoring tools for each assessment [70–72, 79]. Participant preintervention scores were compared with population means. Postintervention scores were compared with preintervention scores. Paired *t*‐tests [82] were used to examine changes between preassessments and postassessments, and effect sizes were examined [83]. Adjustments were not made for multiple comparisons because of the exploratory nature of the study [73]. If missing data were to occur, the results for the corresponding assessment would not be included in the overall assessment and evaluation of the 8‐week program.

## 3. Results

### 3.1. Demographics

Eleven autistic adults (five men and six women) between 25 and 48 years of age enrolled in the study. Ten completed the entire program and one dropped out before the start date because of a health complication. Ten participants completed all enrollment steps and met via Zoom to complete their verbal consent. Participants included six women and four men, 60% (*n* = 6) identified as white, 60% (*n* = 6) were employed, and half (*n* = 5) reported being single. Five participants selected the afternoon session (four women and one man) and five selected the evening session (two women and three men). Eight participants resided in the United States, one in Ireland, and one in Canada. Participants were separated evenly into two groups based on availability and both groups received the same intervention, group session content, and homework each week. This study had no control group. All participants in the current study had some previous knowledge about interoception and reported having a diagnosis of autism as well as interoceptive differences. All participants had prior knowledge of interoception.

### 3.2. Participation

Of the 10 participants who completed the entire program, eight attended all eight sessions and two attended five of the eight sessions. At the start of the program, all 10 participants were offered weekly coaching sessions. The sessions were intended to support participants during weekly homework and provide additional education to participants. Seven out of the 10 participants participated in coaching sessions ranging from one to eight coaching sessions and were typically 30 min long (see Table 1). Researchers split coaching of participants between themselves based on participant availability, and each researcher utilized the weekly homework to guide their coaching sessions. Lack of participation in coaching by three of the participants was due to scheduling, level of interest in the coaching session, or participants′ personal preference for amount of time spent engaging in program activities. We did not experience any technical issues or adverse events for the duration of the program.

**Table 1:** Coaching participation.

Number of coaching sessions attended	Number of participants
8	2
6	1
4	1
3	1
1	2
0	3

### 3.3. Comparison to General Population

We first compared participant scores with general population scores for interoception, anxiety, alexithymia, and QoL to assess areas of difficulty experienced by the participants. The majority of participants scored one or more standard deviations higher than the mean *t*‐score for interoception on the ISQ‐8 at pretest, with a mean score of almost one standard deviation higher (population mean = 50, SD = 10, participant mean = 59.0), indicating weaker interoception than the population mean. GAD‐7 mean score was in the moderate level of anxiety at pretest. Participants′ mean pretest scores were more than one standard deviation above the mean *t*‐score on the GAFS‐8 (alexithymia), indicating a greater amount of alexithymia than the general population. Participants scored one or more standard deviations below the mean in the physical, psychological, and environmental domains at pretest, indicating lower QoL than the general population in those three categories [84]. The social domain mean was less than one standard deviation below the population mean, indicating lower QoL than the general population, but not as low as other categories.

*Aim 1* examined feasibility of an 8‐week online interoception pilot study for autistic adults. Program evaluations were completed in Weeks 4 and 8. Seven participants completed the program evaluation at midterm and all 10 completed it at the end of the program. Participants provided responses to open‐ended, multiple‐choice, and “yes” or “no” questions on the survey. See Appendix 4 for the list of questions.

Program evaluation feedback was predominantly positive. See Table 2 for participant comments. All participants (100%) responded that they would recommend this program to other autistic adults at midterm and final program evaluations. At midterm, 85.7% found overall program and group session length to be a good match for them. At postintervention, 80% supported the program and group session length. One participant noted that having sessions twice a week with coaching in between would have been helpful, whereas some expressed a preference for shorter sessions. The online delivery of the program was generally well received, aligning with participants′ learning styles. Midterm results showed that 42.9% of participants responded “somewhat true” when asked if they felt more confident in understanding their body signals. However, at the end of the program, 40% responded “true” and 30% responded “very true” with an overall 70% experiencing increased confidence. All respondents found that the weekly sessions helped to increase their understanding of interoception with 10% reporting “somewhat true,” 60% “true,” and 30% “very true” at the end of the program. Comments in response to what was the most helpful part of the program included “I didn′t realize how vast the area of interoception was, and I′m so grateful to have learned this,” “It was helpful to hear others describe things that I experience as well but not always able to describe in a way that others can understand,” and “Being forced to consider my feelings in different contexts and form a reading of them. Also, reinforcement that all interpretation of feelings is valid.” One participant said the most helpful part of the program was “learning about each other′s strengths and weaknesses and how we′re all different.”

**Table 2:** Participant comments from the program questionnaire.

What was the most helpful part of the program?	“Feedback from instructors.” “Learning about each other′s strengths and weaknesses and how we are all different.” “The discussion.” “It was helpful to hear others describe things that I experience as well but not always able to describe in a way that others can understand.” “Being forced to consider my feelings in different contexts and form a reading of them. Also, reinforcement that all interpretation of feelings is valid.” “Me having the coaching session and learning other ways to deal with anger.” “The trainers and their knowledge and sharing their own experiences.” “Step‐by‐step instructions.”

What changes would you recommend?	“Simpler wording.” “More individualization in the assignments…more personal problem solving instead of more general experiments.” “Longer sessions with more time between meetings. Possibly more focus on emotional/mental health.” “I think video demonstrations of activities for homework might be very helpful for those of us that are more visual learners.” “A bit more discussion time and a bit less experiments during group. (Not zero experiments, just less.) Might be helpful to do a couple of experiments in coaching session and then report/discuss our experiences together in a group.”

Additional comments	“The OT students did a great job of getting discussion flowing when the participants were hesitant: So glad they did because there was so much great discussion once the ‘floodgates’ would open, which often means a member of the OT team opening that gate ” “I think it was great to have so many instructors – it decreased pressure on participants to answer/talk in the moment which can be difficult.” “I did not realize how vast the area of interoception was, and I′m so grateful to have learned this.” “I had a bit of background knowledge of interoception coming into this, but it was helpful to try exercises I had not thought of.” “Actually exceeded my expectations.”

*Note:* All quotes received from participants are included in this table.

Zoom coaching was considered valuable by participants who attended them. One participant said the most helpful part of the program was “me having the coaching session and learning other ways to deal with anger.”

The instructor‐to‐participant ratio and the online delivery were rated positively. One participant stated, “I think it was great to have so many instructors – it decreased pressure on participants to answer/talk in the moment which can be difficult.” Materials, topics, and homework covered in the sessions were seen as relevant and useful for enhancing participants′ understanding of interoception. The instructors′ knowledge and preparation were positively evaluated by the participants and the hands‐on experiences conducted during sessions were found to be valuable. The activities and strategies taught were considered helpful for improving participants′ interoceptive experiences, and many participants expressed an intention to utilize what they learned in their daily lives. Participant #1 suggested having “More individualization in the assignments…more personal problem solving instead of more general experiments,” having “Longer sessions with more time between meetings. Possibly more focus on emotional/mental health.” Participant #2 suggested “I think video demonstrations of activities for homework might be very helpful for those of us that are more visual learners.” Participant #3 suggested “A bit more discussion time and a bit less experiments during group. (Not zero experiments, just less.) Might be helpful to do a couple of experiments in coaching session and then report/discuss our experiences together in a group.”

### 3.4. Post Intervention Changes

*Aim 2* examined quantitative changes after participation in the program. All mean scores from the ISQ‐8 (interoception), GAFS‐8 *t*‐scores (alexithymia), WHOQoL‐BREF (QoL) domains, and GAD‐7 (anxiety) showed improvement, although insignificant, between pretests and posttests. See Table 3 for details. Small effect size improvements were reported in interoception (*d* = 0.34) and social and environmental QoL (*d* = 0.28 and 0.45, respectively; [83]). Medium effect size improvements were reported in alexithymia (*d* = 0.55) and physical QoL (*d* = 0.68). A large effect size improvement was reported in psychological QoL (*d* = 0.83). There were no missing data on any preanalyses or postanalyses results.

**Table 3:** Post intervention changes.

Test (standard mean and SD)	Premean (SD)	Postmean (SD)	*p*	*d*
ISQ‐8 T‐score (0–100, SD = 10)	59.00 (3.51)	56.88 (5.84)	0.36	0.34
GAD‐7 (0–21, no standard scores)	13.63 (4.75)	12.87 (4.58)	0.43	0.08
GAFS‐8 T‐score (0–100, SD = 10)	63.95 (7.42)	58.40 (11.75)	0.08	0.55
WHOQoL‐BREF				
Physical (0–100, population mean = 73.5, SD = 18.1)	48.5 (11.90)	57.1 (11.74)	0.09	0.68
Psychological (0–100, population mean = 70.6, SD = 14.0)	52.71 (14.13)	53.57 (7.96)	0.81	0.83
Social (0–100, population mean = 71.5, SD = 18.2)	62.5 (18.58)	64.0 (17.31)	0.45	0.28
Environmental (0–100, population mean = 75.1, SD = 13.0)	61.0 (24.84)	67.13 (16.97)	0.24	0.45

Abbreviations: GAD‐7, general anxiety disorder; GAFS‐8, general alexithymia factor score; ISQ‐8, Interoception Sensory Questionnaire. SD, standard deviations; WHOQoL—BREF = World Health Organization Quality of Life—BREF.

## 4. Discussion

The intent of this study was to examine whether it is feasible to conduct an online program to address interoception in autistic adults while determining the appropriateness of the evaluation tools used and testing the intervention on a small number of participants before conducting a larger study using the intervention.

### 4.1. Comparison to General Population

Participants in this study had differences in interoceptive awareness compared with the general population, but this was expected due to the fact that each had some experience with interoception knowledge because they responded to the recruitment requests, so it may not have represented a pattern in the general population of autistic adults. The moderate level of anxiety seen in this group was consistent with previous studies of autistic adults [31]. Most individuals in the current study reported high to very high levels of alexithymia at the start of the program, consistent with previous literature [70, 85, 86]. A total of 80% of participants reported improved posttest alexithymia. Lower QoL than the general population is also consistent with previous studies [15, 47].

### 4.2. Participation

All participants made some improvement from baseline to the end of the program, no matter if they missed sessions or not. Based on assessment results, coaching sessions appeared to support participants′ engagement with the program content and to help improve their interoception abilities throughout the program. However, participants who did not attend coaching sessions also demonstrated improvements in interoceptive awareness without significant differences. These results suggest that both group participation and group participation with coaching sessions supported improved interoceptive abilities in autistic adults. It is possible that coaching may be more beneficial to some than to others, depending on their preferences for social interaction and ability to work independently.

### 4.3. Feasibility

The present study provides the first published evidence suggesting feasibility of an online interoception‐based occupational therapy program. Feasibility questions included whether this type of program would be acceptable to adults, whether the online format would be acceptable to participants, whether they could gain knowledge from this format, whether they would sustain attendance for the 8 weeks of the program and whether they would find the coaching sessions valuable for their learning experiences. Findings indicate that this is a feasible intervention for autistic adults. Scores for interoceptive awareness, alexithymia, anxiety, and QoL in these autistic adults showed insignificant improvements in each area with a range of small to large effect size over the 8 weeks. The sample size was small, but observed improvements in all mean scores were encouraging. Sample size is generally not considered as important for feasibility or pilot studies as the focus is not on the statistical outcomes, but on the feasibility of the intervention so this is not considered a major weakness [87].

All participants reported engagement in this program as valuable and continued attendance for the 8 weeks of the program. Participants provided insightful recommendations for improvements, including more individualization in assignments, increased focus on emotional/mental health, and enhancements in visual learning materials. Program evaluation results about Zoom coaching sessions indicate that group participation with and without coaching sessions improved interoceptive abilities. The instructor‐to‐participant ratio was perceived as favorable, and the online delivery format was well received, which may suggest the small group online format for online discussion was a positive environment for participants that was beneficial for the group discussions. The participant feedback survey provided important considerations for future replications of this program in addition to consideration for providing other forms of occupational therapy interventions to support autistic adults.

### 4.4. Post Intervention Changes

Improvements were seen postintervention but were insignificant in all assessed areas. This lack of significant changes is possibly due to the small sample size, since effect sizes ranged from small to large. It may also be from the relatively short 8‐week program or lack of assessment sensitivity. Seeing improvement in interoception using the *Interoceptive Curriculum* was consistent with studies of in‐person pediatric populations [9, 52]. It was also consistent with the in‐person adult interoception interventions [58–61]. Improvements seen in the current study are also consistent with findings from Quadt et al. [16], who demonstrated improved anxiety symptoms in autistic adults after interoception interventions with the number of scores in the severe anxiety range decreasing by 20% and those in the mild anxiety range increasing by 20%. Improvement was not as large as those seen in the pediatric population and the other adult programs. This may have been a function of a small sample size, lack of in‐person interaction, use of self‐report, lack of assessment sensitivity, or shorter intervention compared with most other programs. Adjustments made for an adult population seemed to be well‐received, according to the program evaluation results.

With minimal published research available on interoception programs for autistic adults, this study has contributed to examining implementation of interoception programs for autistic adults. Following participation in the current program, 70% of participants reported improvements in interoception, which provides encouraging data supporting the possibility of using this occupational therapy‐based interoception intervention for autistic adults. All participants in this study reported previous experience and knowledge about interoception, which may have limited the potential for improvement.

### 4.5. Strengths and Weaknesses

A strength of the current study is that it was possible to recruit participants from across the United States and from two other countries without the need for physical proximity to have access to the program. The ability to reach individuals in a wide geographical area in an online format could allow future programs to expand even further and potentially reach underserved geographical locations. Although geographical diversity was seen in this study, other participant characteristics revealed a predominantly female sample, with the majority in the 30–39‐year age range. Most participants identified as white, employed, and single. It is important to consider these demographic characteristics when interpreting the findings to understand their potential for generalizability to a larger autistic adult population. Translators or translation software, in addition to translating the written program material, could be added to the study team to include non‐English speakers. Increased use of coaching sessions could address the needs of those with different literacy levels.

The use of the program evaluation feedback with this group of adults provided an opportunity to actively incorporate the lived experience of the study participants, more than is possible in programs for children. It fostered a flexible and collaborative approach that was responsive to the clients′ individual needs, which is supported by other autism research [78].

All study participants had previous interoception knowledge, which may have impacted the outcomes. The online format allowed for greater diversity among participants who were able to engage in the program and was endorsed as the preferred method of interaction among the participants. Many participants found the Zoom setting to be well suited to this program due to the ability to participate from a familiar environment, turn off the camera when desired, and participate in written format via the chat. The empowering and adaptive approach in this program may have allowed participants to explore interoception in a safe environment with adaptations to best meet their needs.

A small sample size and no control group limited the strength of the findings and the ability to examine statistically significant relationships and changes from participation in this program. In addition, the trustworthiness of responses to the self‐report questions in each assessment limited the validity of the responses, possibly being subject to participant response bias and survey design errors. Because the program evaluation questions were generated by the research team, their psychometric properties have not been examined so design errors may be present. Self‐report questions such as those used in the measures for interoception, alexithymia, anxiety, and QoL were rated on a scale and may be less trustworthy than observational or physiological response data since interpretation of answer choices may differ between participants. However, a person′s understanding of their own unique experience seems key to understanding interoceptive sensibility, and information from autistic people about their own experience of autism has been identified as a largely untapped resource for enhancing clinical and research tools [88]. We, therefore, believe self‐report to be appropriate and a strength of this study. Additional physiological assessments would add to the strength of the findings, but this was not an option in the online format.

The demographic diversity (gender, income level, and education level) of our sample is not likely to be representative of the autistic adult population, but the results from this study still add valuable information to the current body of literature given the current lack of research available on the efficacy of interoception interventions [15]. Moreover, the inclusion criteria limit the representation of autistic adults who may rely on a caregiver for communication assistance.

### 4.6. Future Direction and Implications

Further research is needed to broaden the body of current evidence on this topic. Future studies need to include a control group to reduce potential bias based on the impact of other extraneous factors and to examine long‐term effects of improved interoceptive awareness. Future studies with larger and more diverse samples are needed to validate the current results. The results emphasize the importance of developing programs that support interoception learning and its impact on other factors, including overall well‐being. Since research in this area is still limited, it would be important to continue studying how interoception can be used to improve anxiety symptoms and the role interoception may play in emotional regulation and mental health. It may be beneficial to add an assessment to quantify emotional regulation or examine a longer program for future studies to allow for more time to learn and apply the program materials. Moreover, more time would allow for a more comprehensive breakdown and explanation of the information and make space for more comprehensive content to be taught within the program.

## 5. Conclusions

This study evaluated the feasibility of an online interoception intervention with autistic adults aged 25 years and older. Interoception strategies were adapted from *The Interoception Curriculum* [56] to be used in an 8‐week online format. The program participants engaged in weekly hour‐long online sessions that included interoception experiments targeting specific body parts, with the intention of improving interoception, anxiety, alexithymia, and QoL. Participants rated the program positively and experienced improvements in all areas, supporting feasibility. Participant feedback was a valuable aspect of this study, allowing the researchers to actively incorporate the lived experience of the study participants. Further research with larger samples and rigorous study designs is needed to validate the findings from this study and to determine effectiveness.

## Author Contributions

S.G., E.O., V.A., K.M., K.R., and C.H. contributed to project design. S.G., E.O., V.A., and K.M. contributed to participant recruitment. S.G., E.O., V.A., K.M., and C.H. contributed to intervention. S.G., E.O., V.A., and C.H. contributed to data analysis. S.G., E.O., V.A., K.M., C.H., K.R., and L.P. contributed to manuscript preparation. C.H., K.R., K.M., and L.P. contributed to project development.

## Funding

No funding was received for this manuscript.

## Conflicts of Interest

Kelly Mahler receives royalties from the Interoception Curriculum.

## General Statement

*Patient and Public Involvement (PPI)*. An autistic occupational therapist advisor was engaged and compensated as a collaborator and contributed to research question development, co‐creation of intervention procedures and study materials, and interpretation of findings, reflecting active patient and public involvement. No third‐party services were involved in the research.

## Figures and Tables

**Figure A1:**
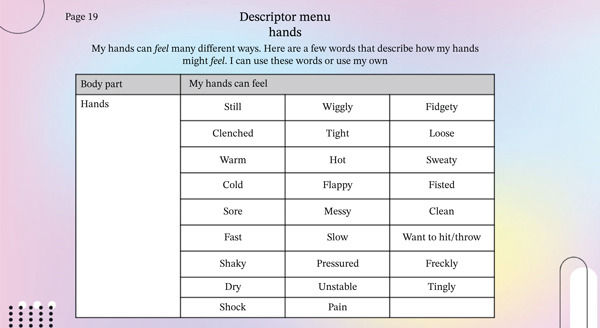
Workbook Example.

**Figure A2:**
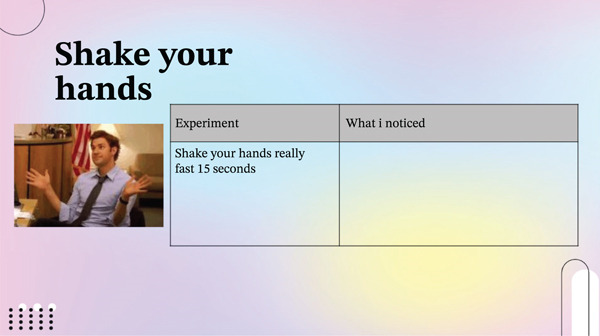
Examples from PowerPoint.

## Data Availability

The data that support the findings of this study are available on request from the corresponding author. The data are not publicly available due to privacy or ethical restrictions.

## Appendix 1: Workbook Examples

**Table A1:** Descriptor menu:Hands—My hands can feel many different ways. Here are a few words that describe how my hands might feel. I can use these words or use my own.

Body part	My hands can feel ____		
**Hands**	Still	Wiggly	Fidgety
	Clenched	Tight	Loose
	Warm	Hot	Sweaty
	Cold	Flappy	Fisted
	Sore	Messy	Clean
	Fast	Slow	Want to hit/throw
	Shaky		
		

## Appendix 3: Interoception Experiments Examples

**Table A3:** Interoception Experiment Example.

Experiment	Where I noticed it	What I noticed
10 Chair pushes		
Eye track a fast‐moving finger		
Take a sip of your beverage		
Hum for 10 s		

*Note:* It is okay to not know how you feel. There is no wrong way to feel. Your inner experience is correct and valid.

## Appendix 4: Interoception Program Evaluation Questions

**Table A4:** Program Evaluation Questions.

Question	Response option
1. The 1‐hour length of each session was a good match for me. **Purpose:** To determine if participants were satisfied with the length of each session or if they would have liked the time to increase or decrease.	It was a good length; I wish they were longer; I wish they were shorter
2. The 8‐week length of the program was a good match for me. **Purpose:** To determine if the participants were satisfied with the length of the program overall.	It was a good length; I wish it was longer; I wish it was shorter
3. Activities were helpful to improve my interoceptive experience. **Purpose:** To make sure that participants found that activities were clearly related to the session and had a purpose.	Not at all true, A little true, Somewhat true, True, Very true Scale of 1–5
4. I will use some of the strategies I learned in this program **Purpose:** To gain an understanding of if strategies will be applicable to participant’s daily life following the programs.	Not at all true, A little true, Somewhat true, True, Very true Scale of 1‐5
Additional Comments:	Type
5. Instructors were knowledgeable and well‐prepared for each session **Purpose:** To make sure we demonstrated professional behavior throughout our program.	Not at all true, A little true, Somewhat true, True, Very true Scale of 1–5
6. The hands‐on experiments used during the sessions were helpful. **Purpose:** To make sure participants felt that the materials used were beneficial and supportive of their learning.	Not at all true, A little true, Somewhat true, True, Very true Scale of 1–5
8. Coaching in between Zoom sessions was helpful **Purpose:** To determine if coaching sessions were beneficial for participants and if they should be continued in future programs.	Not at all true, A little true, Somewhat true, True, Very true Scale of 1–5
9. The instructor‐to‐participant ratio during meetings was a good balance? **Purpose**: to determine if the class size was effective to give each participant the about of support from instructors and social participation with other participants.	Not at all true, A little true, Somewhat true, True, Very true Scale of 1–5
Additional Comments:	Type
10. As a result of this program, I feel more confident in my understanding of my body signals **Purpose:** To determine if clients feel that they are better able to identify interoception and features of interoception than before they began the program.	Not at all true, A little true, Somewhat true, True, Very true Scale of 1–5
11. The materials used in the weekly sessions were useful for improving my understanding of interoception **Purpose:** To make sure the contents of the sessions were purposeful to participants and helped to improve their understanding of interoception.	Not at all true, A little true, Somewhat true, True, Very true Scale of 1–5
12. The topics covered in the weekly Zoom sessions were relevant to me **Purpose:** To make sure that clients found the content to be relevant and meaningful.	Not at all true, A little true, Somewhat true, True, Very true Scale of 1–5
13. The online delivery of this program was a good match for my learning style. **Purpose:** To understand whether or not the Zoom format was appropriate for the learning styles of each participant.	Not at all true, A little true, Somewhat true, True, Very true Scale of 1–5
14. Overall, this program satisfied my desire for increasing my understanding of my interoception experience. **Purpose:** To determine if the program improved participants′ knowledge of interoception and if they felt that the program provided enough information on interoception.	Not at all true, A little true, Somewhat true, True, Very true Scale of 1–5
15. Overall, the homework assignments were helpful to my understanding of interoception. **Purpose:** To determine if the homework was helpful for the participants learning and understanding of interoception.	Not at all true, A little true, Somewhat true, True, Very true Scale of 1–5
16. This program matched what I thought I was signing up for. **Purpose:** To determine if the level of communication prior to the start of the program was adequate to help participants understand what they were committing to.	Not at all true, A little true, Somewhat true, True, Very true Scale of 1–5
Additional Comments:	Type
17. What was the most helpful part of this program? **Purpose:** To gain a subjective understanding of the benefit for each individual participant.	Type
18. What changes would you recommend? **Purpose:** To determine areas of improvement for future programs.	Type
19. I would recommend this program to other autistic adults. **Purpose**: To determine if participants feel that this program is beneficial and fills a need for other autistic adults.	Yes, no
20. I would be open to an interview to share more thoughts about this program. **Purpose:** To assess if participants would be willing to provide more in‐depth feedback about the program.	Yes, no
Additional Comments:	Type
